# The design and development of a hybrid off-job crafting intervention to enhance needs satisfaction, well-being and performance: a study protocol for a randomized controlled trial

**DOI:** 10.1186/s12889-020-8224-9

**Published:** 2020-01-28

**Authors:** Merly K. Kosenkranius, Floor A. Rink, Jessica de Bloom, Machteld van den Heuvel

**Affiliations:** 1 Department of HRM & OB, Faculty of Economics and Business, University of Groningen, Nettelbosje 2, 9747 AE Groningen, the Netherlands; 2 School of Social Sciences, Tampere University, Kalevantie 5, 33100 Tampere, Finland; 3 Department of Work and Organizational Psychology, University of Amsterdam, Nieuwe Achtergracht 129B, 1018 WS Amsterdam, The Netherlands

**Keywords:** Off-job crafting, Psychological needs, Well-being, Job performance, M-health

## Abstract

**Background:**

Employees dealing with job demands such as high workload and permeable work-life boundaries could benefit from bottom-up well-being strategies such as off-job crafting. We have developed a hybrid off-job crafting intervention to promote off-job crafting, a proactive pursuit to adjust one’s off-job time activities to satisfy one’s psychological needs. This hybrid intervention contains both on-site (two trainings) and online elements (smartphone app) to enhance employees’ well-being and performance within different life domains.

**Methods:**

The study is designed as a randomized controlled trial with an intervention group and a waitlist control group. The study population will be Finnish knowledge workers. The intervention program focuses on six psychological needs (detachment, relaxation, autonomy, mastery, meaning, and affiliation) proposed by the DRAMMA model. The intervention will consist of the following components: 1) an on-site off-job crafting training, 2) an individual off-job crafting plan for the four-week intervention period, 3) Everydaily smartphone app usage, and 4) a training session for reflection. The study outcomes are assessed with online questionnaires once at baseline, weekly during the intervention period and twice after the intervention (two-week and six-week follow-up). Moreover, during the second training session, participants will participate in a process evaluation to shed light on the mechanisms that can affect the effectiveness of the intervention.

**Discussion:**

We expect that the intervention will stimulate off-job crafting behaviors, which may in turn increase well-being and performance in both non-work and work domains during and after the intervention (compared to baseline and to the control group). The intervention may provide employees with additional resources to deal with various stressors in life. Furthermore, this off-job crafting intervention could also offer performance benefits for the employers such as increased organizational citizenship behaviors among employees.

**Trial registration:**

The Netherlands Trial Register (NTR): NL8219, December 9, 2019. Registered retrospectively. https://www.trialregister.nl/trial/8219

## Background

Demographic and economic changes have increased the need for employees to stay in the workforce longer than before, while also retaining high well-being and productivity. At the same time, the current workforce has to deal with job demands such as high workload and permeable work-life boundaries [1]. Excessive job demands can turn into stressors, which over time can lead to serious health outcomes, such as exhaustion [2]. This, in turn, can lead to negative consequences on the organizational level, such as increased absenteeism rates [3].

Scholars have proposed that employees dealing with high job demands could benefit from bottom-up well-being approaches [4, 5]. One of such approaches is job crafting, which refers to proactively adjusting one’s level of job demands and resources at work [6]. Although research has demonstrated that proactively adjusting one’s work activities to satisfy psychological needs can help employees to achieve higher well-being at work [7], limited research exists on the benefits of proactively adjusting one’s off-job time activities to satisfy psychological needs during non-work time, referred to as off-job crafting.

Within non-work domain, leisure crafting was first proposed as a concept different from mere participation in leisure activities [8], and later defined as “the proactive pursuit and enactment of leisure activities targeted at goal setting, human connection, learning and personal development” [9]. As people might engage in both recreational activities (e.g., sports, hobbies), and other non-work time activities (e.g., childcare, domestic tasks, volunteer work) to satisfy their psychological needs during the time they are not working, we refer to this form of crafting as off-job crafting. Off-job crafting may help to recover from stressful work because the demands on person’s psychophysiological systems are lower during that time and psychological resources can be replenished [10].

According to self-determination theory [11] all individuals have three innate psychological needs: autonomy, competence, and relatedness. Humans who actively seek out opportunities that satisfy these psychological needs will, in turn, experience positive psychological outcomes such as higher well-being [11] and performance [12]. More recently, Newman, Tay, and Diener [13] proposed a model of six psychological mechanisms mediating the relationship between leisure and subjective well-being. According to the DRAMMA model, these six psychological needs are: detachment, relaxation (referred to as “recovery” in the original model), autonomy, mastery, meaning, and affiliation. Detachment is characterized by mentally disengaging from work-related matters, while relaxation refers to low levels of mental or physical activation and little physical or intellectual effort [14]. Autonomy, one of the basic psychological needs proposed by Ryan and Deci [15], is the desire to experience ownership of one’s behavior. Mastery involves seeking learning opportunities and optimal challenges to experience feelings of achievement and competence [14]. Meaning refers to engaging in activities that individuals perceive as opportunities to gain something valuable in life [16]. Affiliation is the desire to experience relatedness and belongingness with other people [15].

Off-job crafting is primarily expected to satisfy psychological needs in non-work related life domains, but due to the universal nature of people’s basic psychological needs, it should also generate indirect spillover effects on work-related well-being and performance [17]. As there are significantly less -or even no- interfering job demands present during non-work time, people should be more effective in rebuilding their psychological resources via off-job crafting. This could ultimately also benefit people at work. We therefore propose that off-job crafting enhances employees’ well-being and performance both within the non-work domain as well as across life domains by the regeneration of psychological resources and satisfaction of psychological needs. To date and to the best of our knowledge, however, interventions to study whether off-job crafting yields these anticipated effects in terms of both well-being and performance in both life domains do not yet exist. We have therefore designed a hybrid off-job crafting intervention to stimulate off-job crafting among employees to enhance their psychological need satisfaction, well-being, and performance.

The aim of this intervention study is to evaluate the effectiveness of a hybrid off-job crafting intervention compared to a waitlist control group on off-job crafting behaviors, psychological need satisfaction, well-being and performance. We hypothesize that off-job crafting can be stimulated in the intervention group compared to a waitlist control group (between-person difference), and that people will engage more often in off-job crafting during and after the intervention period than before (within-person changes). Moreover, we expect that increased off-job crafting will lead to higher well-being and performance during and after the intervention (compared to baseline and to the control group). We hypothesize that as the result of the intervention, participants will report higher basic need satisfaction and satisfaction of psychological needs during and after the intervention compared to baseline and to the waitlist control group. In terms of work-related well-being outcomes, we expect that participants will report higher work engagement and job satisfaction during and after the intervention compared to baseline and to the waitlist control group. In the non-work domain, we hypothesize that participants will report higher subjective vitality, private life satisfaction and health status and lower stress and mental fatigue levels during and after the intervention compared to baseline and to the waitlist control group. We also expect the intervention to have a positive effect on both work-related and non-work performance. Namely, we expect participants to report higher job performance and organizational citizenship behavior at work and increased family role performance in the non-work domain during and after the intervention compared to baseline and to the waitlist control group. Additionally, we will explore, whether variables such as proactive personality, focus on opportunities, selection, optimization, and compensation strategies, need strengths, home and job demands, job crafting and off-job time activities have a role in participants’ engagement in different off-job crafting behaviors and its effects to their well-being and performance in different life domains.

The intervention development is partly based on the Intervention Mapping approach [18] which provides a stepwise process for developing evidence-based health promotion interventions. As a starting point, the planning group, consisting of researchers and well-being trainers, explored the needs and necessity of managing work-life balance and optimizing the use of leisure time, both theoretically and practically in the Finnish context. Recent studies [19, 20] have demonstrated that Finnish workers with knowledge-intensive jobs have difficulties to manage boundaries between work and non-work time. The advancements in mobile technology have made it possible for the employees to choose their work time and place. A study by Ropponen and colleagues [19] demonstrated that majority (79%) of employees felt the need to be available for the work all the time. According to the study conducted by Toivanen and colleagues [20], 42% of employees reported that they think about job-related matters often or rather often at home and only less than third reported feeling that they are well-recovered from work-related stress. Moreover, non-work time itself can also present people with different type of demands. Salmela-Aro and Upadyaya [21] showed that personal demands such as economic problems and taking care of dependents (e.g., children, elderly parents) vary across the lifespan of Finnish employees and are associated to work burnout. Based on the needs assessment, the following program objectives were formulated: increasing employees’ awareness of their psychological needs, and demands and resources both at work and in personal life; increasing awareness of off-job crafting possibilities; and stimulating off-job crafting behaviors among employees (including during breaks at work). We adapted the design of an existing job crafting intervention [22] to achieve these objectives. As part of the off-job crafting intervention, participants will (1) participate in two on-site trainings, (2) follow a self-composed off-job crafting plan, and (3) use the Everydaily smartphone app. The materials were pretested with a small group of students and adjusted based on their feedback. Before delivering the intervention, pilot testing of the program materials took place with employees from a target organization.

## Methods/design

### Study design

**Fig. 1:**
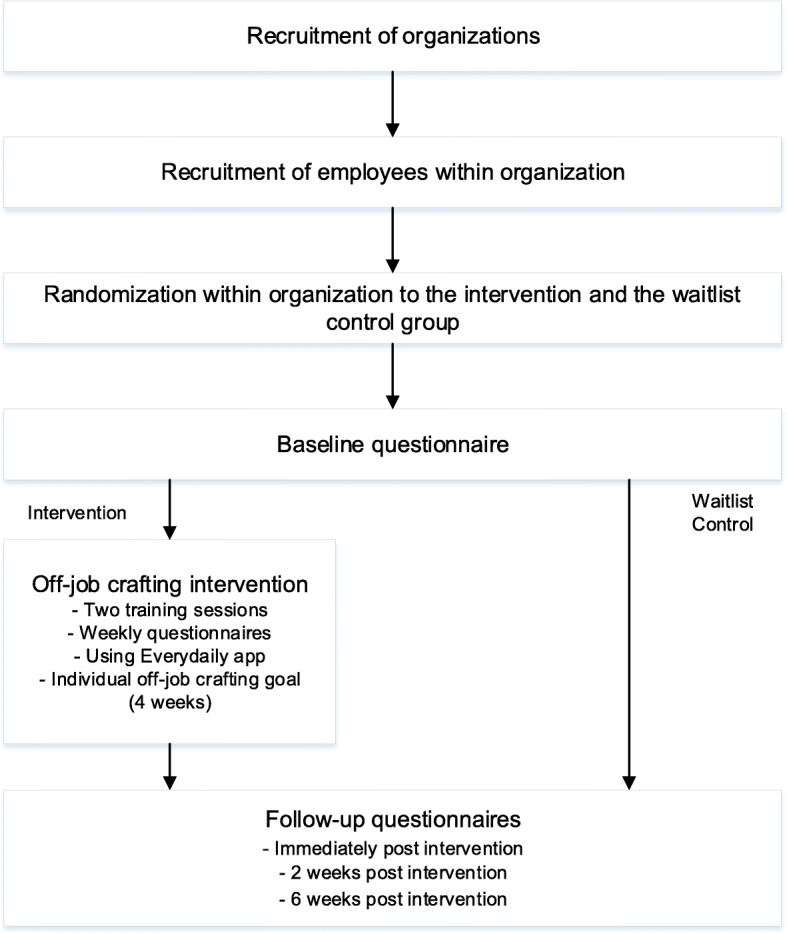
Off-job crafting intervention design

The effectiveness of the hybrid off-job crafting intervention will be evaluated using a two-armed randomized control trial with an intervention group and a waitlist control group. The study design is presented in Fig. 1.

The study will be carried out in Finland from October 2019 to May 2020. All participants who have given informed consent to participate in the study will first fill out an online baseline questionnaire, concerning demographics (e.g., age, gender, education), basic job information (e.g., work hours), off-job and job crafting, psychological needs, well-being (e.g., mental fatigue, work engagement) and performance (e.g., family role performance, organizational citizenship behavior) 2 weeks before the intervention period. Additionally, intervention group participants will receive a small task to complete before the first training session. The task consists of composing a list of things that the participant likes and dislikes doing during their off-job time. In addition, they are asked to take two picture that represent: 1) an activity that they loved doing over the past week and 2) an activity that they did not like to do during their off-job time. One week before the first training session, participants will receive a reminder to complete the questionnaire and the homework task. Two weeks after the baseline measurement, intervention group participants will take part in a half-day long training session. Shortly before the training, each intervention group participant receives a personal feedback report containing information about their baseline scores on DRAMMA needs satisfaction and comparison of their scores to reference values derived from other published scientific studies with Finnish and European knowledge workers as the reference group [23–25]. During the four-week intervention period, every Tuesday, intervention group participants will receive an email with a link to a weekly online questionnaire. In case of non-response, reminders will be sent on Wednesdays and Thursdays. The control group will be asked to fill out a second questionnaire immediately after the end of the intervention period of the intervention group. Additionally, participants from both groups will receive the same online questionnaire via email twice during the post-intervention period (two and six weeks after intervention). After the intervention period, the intervention group will participate in a half-day long reflection session.

After the intervention, the waitlist control group will be offered a chance to participate in the trainings and all the intervention materials will be made available for them, including the booklet and Everydaily smartphone application.

### Study population and recruitment

The study population is recruited from diverse private and public organizations in the capital region and Pirkanmaa region in Finland. The participants are full-time employees with knowledge-intensive jobs. The contacted organizations have previously been interested in Tampere University recovery studies or belong to the network of the trainers delivering the intervention. We will first contact the organizations’ HR representatives by sending them an informational recruitment letter, followed by follow-up calls. Individual informational meetings with interested companies will be arranged.

After the company has agreed to participate in the study, we will forward our recruitment email to their employees. This email includes additional information about the study, including the timeline of the intervention. An organization can take part in the study if there are at least 12 employees interested in participating in the study. In each participating organization, a person will be appointed to facilitate the communication between participants, trainers, and researchers. This person will also provide the employees with a registration form (requesting employee’s name and contact information). Within each organization, we aim to assign participants randomly either to the intervention or to the waitlist control group. After the intervention, employers will receive an anonymized group feedback regarding the development of participants’ levels of stress, mental fatigue, job performance and organizational citizenship behavior across different measurement points.

### Training sessions

The training sessions take place on site for a maximum of 14 employees at a time. Trainers are experienced occupational well-being coaches trained by the researchers. Additionally, they have been actively involved in the intervention development process and are trained in various relaxation methods (e.g., yoga, meditation).

The first training session will focus on introducing the intervention, increasing awareness of stress, psychological needs and off-job crafting, reflecting on personal off-job crafting behaviors in small groups, and developing an individual off-job crafting plan for the intervention period. After a round of introductions, trainers will introduce theoretical findings about stress, off-job crafting and the DRAMMA model. Next, participants will work with their personal feedback reports and homework tasks to reflect on their psychological need satisfaction scores and off-job time activities first individually and then within small groups. The introduction section is followed by several practical exercises.

The practical psychological needs training is divided into six parts, each focusing on a specific DRAMMA need. First, trainers will provide a short theoretical overview of each dimension and a few examples of different strategies found to enhance that specific need. This is followed by a practical exercise addressing the same psychological need. For detachment from work, participants will be asked to think of a transition ritual [26] that would help them to clearly separate their work and non-work time. To introduce a relaxation strategy, the trainers will conduct a short relaxing stretching exercise. To enhance meaning, participants will engage in a short exercise that helps them to become aware of and act in accordance with their own personal values [27]. For mastery, they will engage in an exercise prompting them to remember a past success experience and to share it with another group member to increase participants’ self-efficacy [28]. After the mastery exercise, participants will engage in a strength spotting exercise to enhance affiliation [29]. Participants will take turns in sharing the strengths they have spotted in their colleague based on previously shared success stories to demonstrate how even brief positive interactions can increase the feeling of belonging and positive affect [30]. As participants share a personal story with each other, they get to know each other better and social bonds are strengthened. Finally, to increase autonomy, participants will learn about SMART goal setting [31]. Participants can find all the above described exercises and other training materials in the booklet which they will receive at the beginning of the first training session.

After the psychological needs training, participants will be asked to choose one psychological need that they wish to focus on during the four-week intervention period. While they are free to choose whichever of the six needs they want to work with, we recommend aiming to work with the psychological need that they scored the lowest on. This recommendation is made to support more balanced needs satisfaction across different needs, which has been found to be associated with higher reported well-being [32]. Participants will work in small groups and will be assisted by trainers to come up with their individual SMART off-job crafting goals. In the end, they will write their goals down in their personal booklets. As forming implementation intentions has been shown to improve goal achievement [33], participants will write a postcard for themselves where they outline how implementing their chosen DRAMMA goal will benefit them, what might potentially interfere with achieving the goal and how to overcome these barriers. The postcards will be sent to the participants during the second week of the four-week intervention as a positive reminder to continue working on their goals.

Next, participants will follow an app tutorial outlining the main functions of the smartphone app Everydaily and create their personal off-job crafting projects in the app. At the end of the first training session, trainers will summarize the main points of the training and the next steps, and participants will have a chance to ask questions about both this training session and the intervention in general.

After the four-week intervention period, participants will attend a second half-day training session. During this session, they first fill out the last weekly questionnaire and will receive new individual needs satisfaction scores, which they can compare to their baseline scores. Next, they have a chance to reflect on their experiences by identifying enablers and barriers that affected their off-job crafting during the intervention period. They will then review their previous goals, set future goals, and have the chance to ask questions. Additionally, we will ask participants to fill out a process evaluation questionnaire at the end of the training session to gather information about intervention reach, dose received and participants’ attitudes toward the intervention [34].

Both training sessions will include several short 10-min breaks between different parts of the training. During these breaks, participants can participate in additional exercises. On the first training day, participants can watch a video of natural surroundings to learn about the restorative effects of nature [35] and do a mindfulness meditation exercise. During the second training session, participants are offered to participate in progressive muscle relaxation exercise [36] and in a mandala coloring session with relaxing music [37].

### Everydaily app

During the first training session, participants will be given individual access codes and instructions for using the Everydaily smartphone app. The app content is specifically developed to present participants with short daily activities (Dailys) to support engagement in off-job crafting behaviors. Participants will create a 4-week wellbeing project in the app. Each day, they are presented with three different Dailys, each addressing at least one of the psychological DRAMMA needs (i.e., detachment, relaxation, autonomy, mastery, meaning, affiliation). Participants are encouraged to select and complete Dailys that correspond to the psychological need that they have selected to focus on during the intervention period. Dailys are based on various techniques (e.g., mindfulness meditation, practicing gratitude, goal setting) that target different DRAMMA needs and have been previously shown to improve well-being [38–40]. After participants have completed a Daily, they can upload a picture or short reflection text about the task to the app. Participants’ picture and text submissions remain private and will only be visible to themselves. Additionally, they are asked to rate the level of DRAMMA needs fulfillment that they experienced during completing the Daily. Participants can additionally rate their well-being within the app. They can collect points for completing Dailys and can follow their progress in terms of needs satisfaction and well-being on graphs within the app. They are encouraged to use the gamified app throughout the intervention period.

### Measures

All seven questionnaires will include the following constructs: off-job crafting, subjective vitality, mental fatigue, stress, health status, private life satisfaction, job satisfaction, work engagement, family role performance, job performance, organizational citizenship behavior, basic need satisfaction, satisfaction of psychological needs, home demands, job demands and job crafting. Additionally, the baseline questionnaire will include need strengths, proactive personality, focus on opportunities, Selection, Optimization, and Compensation strategies and background variables. Both the baseline and the fourth weekly questionnaire will additionally measure participation in different off-job activities.

### Off-job crafting

Off-job crafting is measured with a new 18-item off-job crafting scale at the baseline and with a shortened 6-item version during and after the intervention period [24]. Crafting for each of the six DRAMMA needs is measured by three items at the baseline and one item during and after the intervention period. Example items are: “Over the past week, I’ve made sure to detach from work-related thoughts during off-job time” (detachment), “Over the past week, I’ve made sure to experience relaxation of my body and mind during off-job time” (relaxation), “Over the past week, I’ve planned my off-job activities so that I experience control over my life” (autonomy), “Over the past week, I’ve organized my off-job activities so that I put my skills, knowledge or abilities into action” (mastery), “Over the past week, I’ve made sure to experience meaning in my life during off-job time” (meaning), and “Over the past week, I’ve made sure to experience close connections to the people around me during off-job time” (affiliation). Participants are asked to respond on a 5-point Likert scale (1 = never, 5 = very often).

### Well-being

#### Subjective vitality

Subjective vitality is assessed with four items from the Subjective Vitality Scale [41, 42]. Participants are asked to indicate on a 5-point scale how often they felt alive and vital, energetic, having energy and sprit, and looked forward to each new day over the past week. The response scale ranges from 1 (very rarely or never) to 5 (very often or all the time).

#### Mental fatigue

Mental fatigue is measured with four items from the Three-Dimensional Work Fatigue Inventory [43] mental fatigue subscale. The items are adapted to one-week period. An example item is: “Over the past week, how often did you feel mentally worn out at the end of the workday?”. The response scale ranges from 1 (never or almost never) to 5 (very often or all the time).

#### Stress

Stress symptoms are measured with a one-item scale adapted from Elo, Leppänen and Jahkola [44]. First, participants indicate whether they have felt stress symptoms over the past week on a scale ranging from 1 (not at all) to 5 (very much). If applicable, participants are asked to specify in which life domain they experienced this kind of stress within the past week. They can indicate this on a scale from 1 (only during off-job time) to 7 (only at work) with the scale’s middle point indicating stress both in private and work life.

#### Health status

Health status is measured with one item [45]. Participants are asked to assess their current general health status on a scale from 1 (very bad) to 5 (very good).

#### Private life satisfaction

Private life satisfaction is measured with a single item adapted from job satisfaction item: “How satisfied have you been with your private life over the past week?”. In this context, private life refers to everything outside of work context, including family life, leisure activities, domestic chores, hobbies. Participants are asked to indicate their private life satisfaction on a scale ranging from 1 (very dissatisfied) to 10 (very satisfied).

#### Job satisfaction

Job satisfaction is measured with a single item: “How satisfied have you been with your job over the past week?”. A single-item measure has been shown to be acceptable to measure job satisfaction [46]. Participants can indicate their job satisfaction on a scale ranging from 1 (very dissatisfied) to 10 (very satisfied).

#### Work engagement

Participants work engagement is assessed with six items from the Utrecht Work Engagement Scale vigor and dedication subscales [47]. The items are adapted to one-week period. Example items are: “Over the past week at my work, I felt bursting with energy” (vigor) and “Over the past week, I was enthusiastic about my job” (dedication). Participants can choose between seven response options from 0 (never) to 6 (always).

### Performance

#### Family role performance

Participants’ performance in private life is assessed with a family role performance scale developed by Chen and colleagues [48]. The 4-item subscale measuring relationship performance in one’s family life is used in this study. The items are adapted to one-week period. An example of relationship performance item is: “Providing emotional support to your family members”. Participants can indicate to what extent their feel that they fulfilled what was expected of them in relation to different aspects of their current family life on a scale from 1 (did not fulfill expectations at all) to 5 (fulfilled expectations completely).

#### Job performance

To measure job performance, participants are asked to rate their overall work performance over the past week on a single-item measure [49]. The scale ranges from 1 (the worst job performance a person could have at your job) to 10 (performance of a top worker at your job).

#### Organizational citizenship behavior

Organizational citizenship behavior is measured with five items from a scale developed by Lee and Allen [50] and an additional item by Goodman and Svyantek [51]. The first three items measure behaviors directed towards the individuals. An example item is: “You have assisted others with their duties”. The next three items measure behaviors directed towards the organization, such as: “You offered ideas to improve the functioning of the organization”. Participants are asked how often they have engaged in these behaviors over the past week on a scale from 1 (never) to 7 (always).

### Potential moderators, mediators and control variables

#### Need strengths

Need strengths is measured with the baseline questionnaire. The items are adapted from Chen and colleagues [25]. In the current study, we use one question per dimensions for detachment and relaxation needs and two questions per dimension for autonomy, mastery, meaning, and affiliation needs. Example items are: “It is important to me to mentally disengage from my work during my off-job time” (detachment), “It is important to me to relax after my work is done” (relaxation), “It is important to me to feel in control” (autonomy), “It is important to me to develop my skills and abilities” (mastery), “It is important to me to achieve a sense of purpose in what I am doing” (meaning) and “It is important to me to experience close connections to the people around me” (affiliation). The need strength is measured on a scale from 1 (not important at all) to 5 (very important).

#### Basic need satisfaction

Basic need satisfaction is measured with nine items from the Basic Psychological Need Satisfaction and Frustration Scale [25]. The scale is reduced to three items per each psychological need based on the highest factor loadings in the study conducted by Chen and colleagues [25]. The items are adapted to one-week timeframe. Example items are: “Over the past week, I’ve felt a sense of choice and freedom in the things I undertook” (autonomy), “Over the past week, I’ve felt capable at what I did” (competence) and “Over the past week, I’ve felt that the people I care about also cared about me” (relatedness). Participants are asked to respond on a 5-point Likert scale (1 = completely untrue, 5 = completely true).

#### Satisfaction of psychological needs

DRAMMA needs experiences are measured with a 16-item scale. Detachments, relaxation, autonomy and mastery dimensions are each measured with three items from the Recovery Experience Questionnaire [14]. In addition, four additional items were developed to measure meaning and affiliation. Example items are: “Over the past week, during time after work, I forgot about work” (detachment), “Over the past week, during time after work, I kicked back and relaxed” (relaxation), “Over the past week, during time after work, I determined for myself how I will spend my time” (autonomy), “Over the past week, during time after work, I did things that challenge me” (mastery), “Over the past week, during time after work, I experienced meaning in my life” (meaning), “Over the past week, during time after work, I experienced close connections to the people around me” (affiliation). Participants can indicate their agreement with the statements on a scale from 1 (I do not agree at all) to 5 (I fully agree).

#### Home demands

Home demands are measured with the Home Demands Scale [52]. The scale consists of three subscales: quantitative home demands (example item: “How often have you been busy at home over the past month?”), emotional home demands (example item: “How often did emotional issues arise at home over the past month?” and mental home demands (example item “How often did you have to do many things simultaneously at home over the past week?”). The scale used in this study consists of nine items with one original mental demands item excluded. Participants are asked to report their demands in private life over the past week with the answer range from 1 (never) to 5 (very often).

#### Job demands

During each measurement, participants are asked to report their past week’s working hours. Additionally, three types of job demands will be measured during each measurement. Workload is measured with three items from the Quantitative Workload Inventory [53]. An example question is: “How often did your job require you to work very fast over the past week?”. Cognitive job demands are measured with three questions from the Copenhagen Psychosocial Questionnaire [54] and the DISC Questionnaire [55]. An example item is: “How often did your work require that you remember a lot of things over the past week?”. Emotional job demands are measured with three items from the Copenhagen Psychosocial Questionnaire [54]. An example item is: “How often did your work evoke unpleasant feelings over the past week?”. The response scales for all job demand items ranges from 1 (very rarely or never) to 5 (very often or all the time).

#### Job crafting

Job crafting is measured with a 4-item job crafting scale [56]. An example item is: “I change my job so it would better fit with who I am”. Participants are asked to respond on a 5-point Likert scale from 1 (very rarely or never) to 5 (very often).

#### Off-job activities

Off-job activities are measured with a newly developed scale. The scale is composed of various scales which have previously been used to measure participation in different leisure activities [57–61]. Participants can indicate the frequency of engaging in different leisure activities (e.g., active socializing, cultural activities, outdoor activities) on a scale from 1 (almost never) to 5 (several times a day).

#### Proactive personality

Proactive personality is measured only with the baseline questionnaire, using a six-item version of the Proactive Personality Scale [62]. An example item is: “I excel at identifying opportunities”. Employees can choose a response from 1 (totally disagree) to 5 (totally agree).

#### Focus on opportunities

Focus on opportunities is measured in the baseline questionnaire with a four-item scale [63]. An example item is: “My occupational future is filled with possibilities”. Participants can reply on a scale from 1 (does not apply at all) to 5 (applies completely).

#### Selection, optimization and compensation strategies

Selection, Optimization and Compensation strategies are measured in the baseline questionnaire with a twelve-item scale [63]. The scale has four subscales: elective selection (example item: “At work, I concentrate all my energy on few things”), loss-based selection (example item: “When things at work don’t go as well as they have in the past, I choose one or two important goals”), optimization (example item: “At work, I make every effort to achieve a given goal”), and compensation (example item: “When things at work don’t go as well as they used to, I keep trying other ways until I can achieve the same result I used to”). Participants can reply on a scale from 1 (does not apply at all) to 5 (applies completely).

#### Background variables

We ask the participants to state their age, gender, highest level of formal education, family status, living situation, type of work, weekly working hours, and tenure.

### Statistical analyses

#### Sample size calculation

The G*POWER software [64] was used to determine the minimum number of participants necessary to guarantee sufficient power of the study. Assuming an effect size of .25 (medium), alpha = .05 and power = .80, the required sample size is 64 participants per group to determine a significant difference between the intervention and the waitlist control group. Taking into account potential non-response and loss to follow-up, we aim to recruit at least 200 employees to ensure an adequate sample size.

#### Basic analyses

All basic analyses are carried out with SPSS 25 [65]. Two-tailed significance level of < .05 will be considered statistically significant. Baseline characteristics of the participants are analyzed using descriptive statistics. Independent samples t-tests or chi-square tests are applied to check whether randomization was successful or whether there were systematic differences between the two groups. Preliminary analysis will be performed, including calculating descriptive statistics (i.e., means, standards deviations), factor analyses for all the scales, internal reliability calculations (Cronbach’s *α*) and bivariate correlations (Pearson’s *r*) between the study variables.

#### Effect evaluation

We will use multilevel modelling techniques to test the change in outcome variables within subjects across seven measurement points before, during and after the intervention (i.e., within subject effect of participating in off-job crafting intervention). We will use Bliese and Ployhart’s [66] approach to estimate multilevel models in R, using the NLME library written by Pinheiro and Bates [67]. Additionally, we will test how participation in off-job intervention compared to control group affects the change in outcome variables across time (i.e., between subjects’ effect of the intervention compared to control group).

#### Moderator and mediator analyses

For moderator and mediator analysis, we will use the nonparametric resampling method of bootstrapping (with 5000 bootstrap resamples) [68]. Hayes’ PROCESS macro for SPSS is used to run the bootstrapping procedure. Bootstrapping does not assume the sampling distribution of the indirect effect to be normally distributed, it is statistically more powerful, and is suitable to use on small samples [68].

#### Process evaluation

At the end of the second training session, a process evaluation will be conducted to provide insights into the process of the intervention and the mechanisms that might have influenced the effectiveness of the intervention. Participants are asked to indicate the extent that they participated in the intervention, the level of effort invested in following the intervention, and whether they perceived participation in the intervention to be time-consuming. Additionally, we ask them to report their level on enjoyment and satisfaction with the intervention, relevance of the training content, and the type of perceived support that they received from others during the intervention. We also ask participants to assess whether they gained new skills and knowledge from the intervention, and to express their level of satisfaction with the performance of the trainers during on-site trainings. Regarding the smartphone app Everydaily, we ask participants to indicate how many Dailys they have completed and whether they also logged all the completed Dailys to the app to gain better understanding about participants’ app use. Regarding off-job crafting goal setting, participants are asked to indicate the psychological need that they worked on during the intervention period and write down the goal that they set during the first training session. They are then asked to rate their goal attainment during the intervention period on a scale from 0 to 100%. Finally, participants can describe the most important things that they learned during the intervention and provide any additional comments.

## Discussion

This article describes the development and design of a hybrid off-job crafting intervention study aimed at enhancing employees’ off-job crafting behaviors, psychological needs satisfaction, well-being and performance.

### Strengths and limitations

The main limitation of the study is that the intervention includes several on- and offline components. With the two-armed randomized control trial (intervention versus waitlist control), it is not possible to evaluate each specific component separately, and it is therefore difficult to establish which intervention components are the most effective. However, we aim to conduct a thorough process evaluation to gain insights into recruitment, reach, fidelity, participants’ attitudes toward the intervention, context, implementation, dose delivered and dose received, and to link the effect and process evaluation results to better understand the different mechanisms that might have influenced the effectiveness of the intervention [34]. Secondly, as the intervention and the waitlist control group participants are recruited from the same organization and potentially form the same departments, contamination may occur. While the trainings, booklets and smartphone app are first only made available for the intervention group, there is still a chance that colleagues participating in the intervention could share their training materials with the waitlist control group participants. To minimize this risk, intervention participants are specifically reminded not to share the training materials with the control group members until the end of the intervention period. Thirdly, another limitation might be participants’ non-adherence to the instructions or dropping out of the study entirely. In order to avoid these potential complications, we will explain the procedures to the participants carefully both in the informed consent form and during the first training session to remind participants about the importance of following the entire intervention program. We will also send them a mid-intervention postcard reminder and will monitor closely the weekly questionnaire response rates, sending reminders to those who do not fill out the questionnaires by the deadlines indicated in the emails. We will further assess adherence to the intervention program with the process evaluation.

To the best of our knowledge, this is the first intervention study to evaluate the effectiveness of a hybrid off-job crafting intervention to enhance off-job crafting behaviors, psychological needs satisfaction, well-being and performance in different life domains. The study has direct practical implications for employees and their well-being. Participation in the trainings, following an individual off-job crafting plan, and using the smartphone app could potentially offer a brief and accessible way for employees to increase their off-job crafting behaviors, which in turn could enhance psychological needs satisfaction, well-being and performance in different life domains. Besides potential benefits for the employees, the intervention could also benefit the organizations, potentially resulting in healthier and more productive employees. Finally, the intervention content allows plenty of room for customization to meet each participant’s individual psychological needs, and can therefore be offered to workers from different professions, educational levels and life situations.

## Data Availability

Data will be shared after completion of the research project in the Finnish Social Science Data Archive. Data and intervention materials are also available on request from the first author.
